# Brain Microstructural Changes Associated With Neurocognitive Outcome in Intracranial Germ Cell Tumor Survivors

**DOI:** 10.3389/fonc.2021.573798

**Published:** 2021-05-26

**Authors:** Winnie Wan Yee Tso, Edward Sai Kam Hui, Tatia Mei Chun Lee, Anthony Pak Yin Liu, Patrick Ip, Vince Vardhanabhuti, Kevin King Fai Cheng, Daniel Yee Tak Fong, Dorita Hue Fung Chang, Frederick Ka Wing Ho, Ka Man Yip, Dennis Tak Loi Ku, Daniel Ka Leung Cheuk, Chung Wing Luk, Ming Kong Shing, Lok Kan Leung, Pek Lan Khong, Godfrey Chi-Fung Chan

**Affiliations:** ^1^ Department of Paediatrics and Adolescent Medicine, Queen Mary Hospital, Li Ka Shing (LKS) Faculty of Medicine, The University of Hong Kong, Hong Kong, Hong Kong; ^2^ Department of Diagnostic Radiology, Queen Mary Hospital, Li Ka Shing Faculty of Medicine, The University of Hong Kong, Hong Kong, Hong Kong; ^3^ State Key Laboratory of Brain and Cognitive Sciences, The University of Hong Kong, Hong Kong, Hong Kong; ^4^ Laboratory of Neuropsychology, The University of Hong Kong, Hong Kong, Hong Kong; ^5^ Department of Neurosurgery, Hong Kong Children’s Hospital, Hong Kong, Hong Kong; ^6^ School of Nursing, The University of Hong Kong, Hong Kong, Hong Kong; ^7^ Department of Psychology, The University of Hong Kong, Hong, Kong, Hong Kong; ^8^ Institute of Health and Wellbeing, University of Glasgow, Glasgow, United Kingdom; ^9^ Department of Oncology, Hong Kong Children’s Hospital, Hong Kong, Hong Kong; ^10^ Department of Paediatrics and Adolescent Medicine, Hong Kong Children’s Hospital, Hong Kong, Hong Kong

**Keywords:** intracranial germ cell tumor, neurotoxicity, diffusion kurtosis imaging, brain microstructure, cognition, functional outcome

## Abstract

**Background:**

Childhood intracranial germ cell tumor (GCT) survivors are prone to radiotherapy-related neurotoxicity, which can lead to neurocognitive dysfunctions. Diffusion kurtosis imaging (DKI) is a diffusion MRI technique that is sensitive to brain microstructural changes. This study aimed to investigate the association between DKI metrics versus cognitive and functional outcomes of childhood intracranial GCT survivors.

**Methods:**

DKI was performed on childhood intracranial GCT survivors (n = 20) who had received cranial radiotherapy, and age and gender-matched healthy control subjects (n = 14). Neurocognitive assessment was performed using the Hong Kong Wechsler Intelligence Scales, and functional assessment was performed using the Lansky/Karnofsky performance scales (KPS). Survivors and healthy controls were compared using mixed effects model. Multiple regression analyses were performed to determine the effects of microstructural brain changes of the whole brain as well as the association between IQ and Karnofsky scores and the thereof.

**Results:**

The mean Intelligence Quotient (IQ) of GCT survivors was 91.7 (95% CI 84.5 – 98.8), which was below the age-specific normative expected mean IQ (*P* = 0.013). The mean KPS score of GCT survivors was 85.5, which was significantly lower than that of controls (*P* < 0.001). Cognitive impairments were significantly associated with the presence of microstructural changes in white and grey matter, whereas functional impairments were mostly associated with microstructural changes in white matter. There were significant correlations between IQ versus the mean diffusivity (MD) and mean kurtosis (MK) of specific white matter regions. The IQ scores were negatively correlated with the MD of extensive grey matter regions.

**Conclusion:**

Our study identified vulnerable brain regions whose microstructural changes in white and grey matter were significantly associated with impaired cognitive and physical functioning in survivors of pediatric intracranial GCT.

## Introduction

Intracranial germ cell tumors (GCT), while rare in the West, are more common in Asia accounting for up to 15% of primary intracranial tumors in Asian children. Germinomas are the most common subtype of GCT. Despite of the excellent survival rate for intracranial GCT, the relatively large volumes and high doses of radiation required to treat intracranial GCT can impact neurocognitive and functional outcomes. Treatment-related neurotoxicity was shown to be particularly harmful to specific cognitive processes, such as executive functions (1, 2). Furthermore, a recent study reported that the intelligence quotient (IQ) of nearly 20% of pediatric intracranial GCT survivors was borderline or worse (3).

Because of the high survival rates of childhood intracranial GCT, there is growing concern over the long-term neurocognitive outcomes. Among the different types of intracranial germinomas, basal ganglia germinomas tended to result in the worst neurocognitive outcomes (4). It is believed that the deep grey matter of children is more susceptible to oxidative damage. Damage to the basal ganglia and deep brain nuclei can lead to cognitive impairment as well as loss of motor control (5). Majority of basal ganglia germinomas with poor neurocognitive outcomes were also associated with early cerebral atrophy (6). It was proposed that atrophy occurs as a result of tumor involvement of the internal capsule fibers or thalamic ganglion cells, with Wallerian degeneration and subsequent interruption of thalamocortical connections leading to detrimental effects on motor function and functional outcomes (7). It is thus conceivable that the cognitive and functional impairments in intracranial GCT survivors are likely due to both grey and white matter damage.

Numerous neuroimaging studies have investigated the association between brain changes and neurocognitive and functional outcomes in brain tumor survivors (8, 9). However, previous studies suggested that brain volume or volumetric white matter changes might be a poor predictor of neurocognitive deficits in the later life of cancer survivors (9–11). Conventional MRI methods might not be sensitive enough to identify changes that are predictive of neurocognitive dysfunction.

We have previously demonstrated that fractional anisotropy (FA), a diffusion metric commonly obtained from diffusion tensor imaging (DTI), correlated with the cognitive outcomes of medulloblastoma survivors (12, 13). Diffusion tensor imaging is a diffusion MRI technique that measures the diffusion of water molecules, a process that is very sensitive to tissue microstructural changes. Fractional anisotropy and mean diffusivity (MD) are the two most common diffusion metrics obtained from DTI. Notably, neurological/functional impairments were found to be more related to white matter injury, whereas cognitive impairments were associated with both white and grey matter injuries (14). So far, there have been no investigations on the relation between grey matter changes and cognitive outcomes in brain tumor survivors.

In the past few years, diffusional kurtosis imaging (DKI), an extension to DTI, has been shown to be more sensitive to microstructural changes in white and grey matter in adult neurological diseases such as stroke (15, 16), traumatic brain injury (17), and Alzheimer’s disease (18). We therefore hypothesized that diffusion metrics obtained from DKI would be more sensitive to the white and grey matter changes of intracranial GCT survivors, and these metrics might be associated with functional and neurocognitive outcomes. The aim of this study was to investigate whether white and grey matter microstructural changes as measured by DKI were associated with functional and neurocognitive outcomes in childhood intracranial GCT survivors.

## Methods

We conducted a cross-sectional study to determine if changes in brain microstructure would correlate with the neurocognitive and functional outcomes of childhood intracranial GCT survivors with a history of cranial radiotherapy. This study was approved by the ethical committees of the Institutional Review Board of the University of Hong Kong and Hospital Authority Hong Kong West Cluster, Kowloon West and Kowloon Central Clusters, and New Territories East and New Territories West Clusters. Informed written consent was obtained from the parents of participants under the age of 18 years or from participants aged ≥ 18 years. Neurocognitive outcomes were assessed using the Full Scale Intelligence Quotient (FSIQ) and functional outcomes were assessed using Lansky/Karnofsky performance scales.

### Subject Recruitment

We recruited all the survivors of childhood intracranial GCT over the past 20 years from the database of the Hong Kong Pediatric Hematology and oncology study group. Of the 62 GCT survivors, 25 completed the full assessments for this study. The remaining 37 survivors were either lost to follow-up due to changes in contact details/moved abroad, or they declined to join the study due to refusal to have follow-up imaging/busy work or school schedule/not interested in joining the study. All of the participants completed treatment at least 1 year prior, were currently free of the primary disease, and were at least 6 years of age at the time of recruitment. Treatment of intracranial GCT was delivered according to a standardized protocol based on SFOP and COG experience (19, 20). Following the initial diagnosis by tissue biopsy or resection, patients with germinoma were treated by chemotherapy with either BEP (bleomycin, etoposide, cisplatin) or SFOP which consisted of alternated cycle of CE-IE (carboplatin, etoposide, ifosphamide). The dose and volume of radiotherapy was based on the response and site of involvement. Patients with non-germinomatous GCT received chemotherapy as above for six cycles followed by craniospinal radiotherapy and tumor boost. Reassessment by MRI was performed every other chemotherapy cycle and at the end of the treatment (3).

Age and gender-matched healthy children were recruited as controls from volunteers or patients undergoing brain MRI for clinical indications such as headaches, but were later confirmed to have no neurological deficits by clinical examination and MRI.

### Data Acquisition

The MRI was performed using a 3.0 Tesla MRI Scanner (Achieva TX, Philips, Best, Netherland) with an 8-channel head coil. The DKI data were acquired using a Stejskal–Tanner diffusion-weighted spin echo echo-planar imaging sequence with the following parameters: two b values (1000 and 2000 s/mm^2^), 32 diffusion-encoding directions, TR/TE = 2000/72 ms, FOV = 234 x 224 mm^2^, acquisition matrix = 96 x 94, slice thickness = 3 mm (no gap), SENSE factor = 2 (along AP), number of averages = 2, and scan time = 12.8 minutes.

Whole-brain T1-weighted images were acquired using MPRAGE with the following parameters: TR/TE = 7/3.2 ms, flip angle = 8°, FOV = 224 x 224 x 167 mm^3^, acquisition matrix = 224 x 224 x 167, and scan time = 2.7 minutes. Fluid-attenuated inversion recovery (FLAIR) sequence was also acquired with the following parameters: IR/TR/TE = 2800/9600/120 ms, FOV = 230 x 182 mm^2^, acquisition matrix = 192 x 157, slice thickness = 2.5 mm, and scan time = 10.7 minutes.

### Image Post-Processing

Brain parcellation: T1-weighted images were used to partition the brain into 90 cortical and subcortical regions based on the Automated anatomical labeling (AAL) atlas using Statistical Parametric Mapping (version 12; SPM12) (https://www.fil.ion.ucl.ac.uk/spm/software/spm12/) with the following steps: (1) DKI data (average of diffusion-weighted images along all diffusion directions) were first co-registered to T1-weighted images in anatomical space. (2) T1-weighted images were normalized to the ICBM 152 template in standard space. (3) The estimated transformation parameters were subsequently applied to the parametric map of diffusion metrics in anatomical space to ensure they were all in standard space for the subsequent region-of-interest analyses.

Diffusion metrics: The DKI data were first corrected in SPM for any misregistration caused by head motion and eddy currents. Diffusional Kurtosis Estimator (version 2.6; https://medicine.musc.edu/departments/centers/cbi/dki/dki-data-processing) was used to obtain diffusion metrics (21) including mean kurtosis (MK), MD and FA.

Region-based analysis: The diffusion metrics of all 90 cortical and subcortical regions from the AAL, and all major white matter tracts from the Johns Hopkins white matter atlas were measured from all subjects. Measurements were obtained by averaging the diffusion metrics from all the pixels within a given brain region. To avoid partial volume effects from non-brain pixels, the following thresholds were used: 0 < FA < 0.9, 0.2 < MD < 3 um^2^/ms, and 0.3 < MK < 2.

### Neuropsychological Tests and Functional Outcomes

The neuropsychological assessments and functional outcomes were performed within 1 year of the MRI. Full scale IQ scores were obtained using the Hong Kong Wechsler Intelligence Scales for Children (WISC) for subjects aged under 16 years and the Wechsler Adult Intelligence Scale – Revised (WAIS-R) for subjects aged 16 years and over. Functional outcomes were assessed using the Lansky play-performance scales for subjects aged 6 to 15 years and Karnofsky performance scales (KPS) for subjects aged 16 years and over.

### Statistical Analysis

Survivors and healthy controls were compared using mixed effects model to account for extra covariance within each matched pair. Multiple regression analyses were performed to determine the effects of microstructural brain changes of the whole brain as well as each segmental white and grey matter regions on IQ and Karnofsky scores with adjustment of sex, age, radiation dosage, length of time since treatment, and treatment methods. Separate regression analyses were performed for each diffusion MRI metric with each white and grey matter regions from the AAL atlas. Holm’s sequential Bonferroni procedure was used to account for multiplicity due to multiple comparisons. All statistical analyses were performed using the computing environment R (R Development Core Team 2018). P-value < 0.05 was considered statistically significant.

## Results

### Patients, Tumor Characteristics, and Treatment Modalities

All of the 25 GCT patients completed IQ tests and functional outcome assessment, but 5 of them failed the MRI examinations because of claustrophobia or technical problems and were excluded from the analyses. Therefore, 20 patients (**Table 1**) together with 14 age- and gender-matched healthy control subjects were included in the final analyses. Of the 20 survivors, 15 were male and five were female, with a median age at diagnosis of 14.4 years and average length of time since treatment of 6.5 years. There were 13 germinomatous germ cell tumor (GCT) survivors and 7 non-germinomatous germ cell tumor (NGGCT) survivors. Tumor locations included the suprasellar/sellar region (*n* = 7), basal ganglia (*n* = 5), pineal region (*n* = 4), bifocal regions (*n* = 3), and brainstem (*n* = 1). Six patients had surgical resection of the tumor, chemotherapy and radiotherapy, whereas 14 had chemotherapy and radiotherapy only. The average total irradiation dose was 42.16 Gy (range 30 – 54 Gy). The average IQ score of GCT survivors was 91.7 (CI 84.5 – 98.8), which was below the age-specific normative expected IQ in the validated WISC IV or WAIS III (*P* = 0.013). The average KPS score of GCT survivors was 85.5, which was significantly lower than normal (*P* < 0.001). All clinical characteristics are summarized in **Table 1**.

**Table 1:** Clinical Characteristics of GCT survivors.

Subject number	Sex	Age at diagnosis (years)	Length of time since treatment (years)	Types of germinoma	Tumor Loci	Size of GCT (largest length in cm)	Presence of hydrocephalus at presentation	Treatment	Total RT dosage (Gy)	RT field CSI + tumor boost dose (Gy)
1	M	11.7	9.3	GCT	Basal ganglia	0.7	No	C + RT	30.6	CSI (21.6) + TB (9)
2	M	15.1	10.9	NGGCT	Basal ganglia	3.0	No	C + RT	50.0	IFRT
3	M	8.8	1.2	NGGCT	Pineal	1.1	Yes	C + RT	46.8	CSI (30.6) + TB (16.2)
4	M	17.9	3.1	GCT	Others - Bifocal	2.3	No	C + RT	30.0	WVRT + TB
5	M	9.7	8.3	GCT	Basal ganglia	3.0	No	C + RT	50.4	WVRT + TB
6	F	10.7	5.3	NGGCT	Sellar/suprasellar	3.9	No	S, C + RT	54.0	CSI (36) + TB (18)
7	M	17.2	6.8	GCT	Pineal	0.9	Yes	C + RT	30.0	CSI (22.5) + TB (7.5)
8	F	17.5	6.5	GCT	Sellar/suprasellar	3.1	No	S, C + RT	36.0	WVRT + TB
9	F	15.8	3.2	NGGCT	Sellar/suprasellar	2.4	No	S, C + RT	54.0	CSI (36) + TB (18)
10	M	17.3	9.7	GCT	Sellar/suprasellar	1.4	No	C + RT	48.0	WBRT
11	M	13.8	12.2	GCT	Basal ganglia	6.0	No	C + RT	52.2	WBRT
12	F	7.7	7.3	NGGCT	Others - Bifocal	4.2	No	C + RT	45.0	CSI (24) + TB (21)
13	F	9.8	6.2	GCT	Sellar/suprasellar	1.0	No	C + RT	45.0	WVRT + TB
14	M	15.2	9.8	GCT	Pineal	2.7	Yes	C + RT	45.5	CSI (25.5) + TB (20)
15	M	10.9	6.1	GCT	Pineal	2.0	Yes	S, C + RT	45.0	WBRT
16	M	16.7	1.3	GCT	Sellar/suprasellar	4.1	No	C + RT	36.0	WBRT
17	M	17.5	2.5	NGGCT	Others - Brainstem	3.3	No	S, C + RT	54.0	CSI (36) + TB (18)
18	M	10.8	8.2	NGGCT	Sellar/suprasellar	1.3	No	S, C + RT	30.0	WVRT + TB
19	M	17.3	9.7	GCT	Others - Bifocal	4.2	No	C + RT	30.6	CSI (23.4) + TB (7.2)
20	M	10.9	1.8	NGGCT	Basal ganglia	3.5	No	C + RT	54.0	WVRT + IFRT

C, chemotherapy; S, surgery; RT, radiotherapy; GCT, germ cell tumors; CSI, cranial spinal irradiation; IFRT, involved field radiotherapy; WBRT, whole brain radiotherapy; WVRT, whole ventricular radiotherapy; TB, tumor boost.

GCT, Germ Cell Tumor; NGGCT, Nongerminomatous Germ Cell Tumor.

### Diffusion Metrics of White Matter

**Figure 1:**
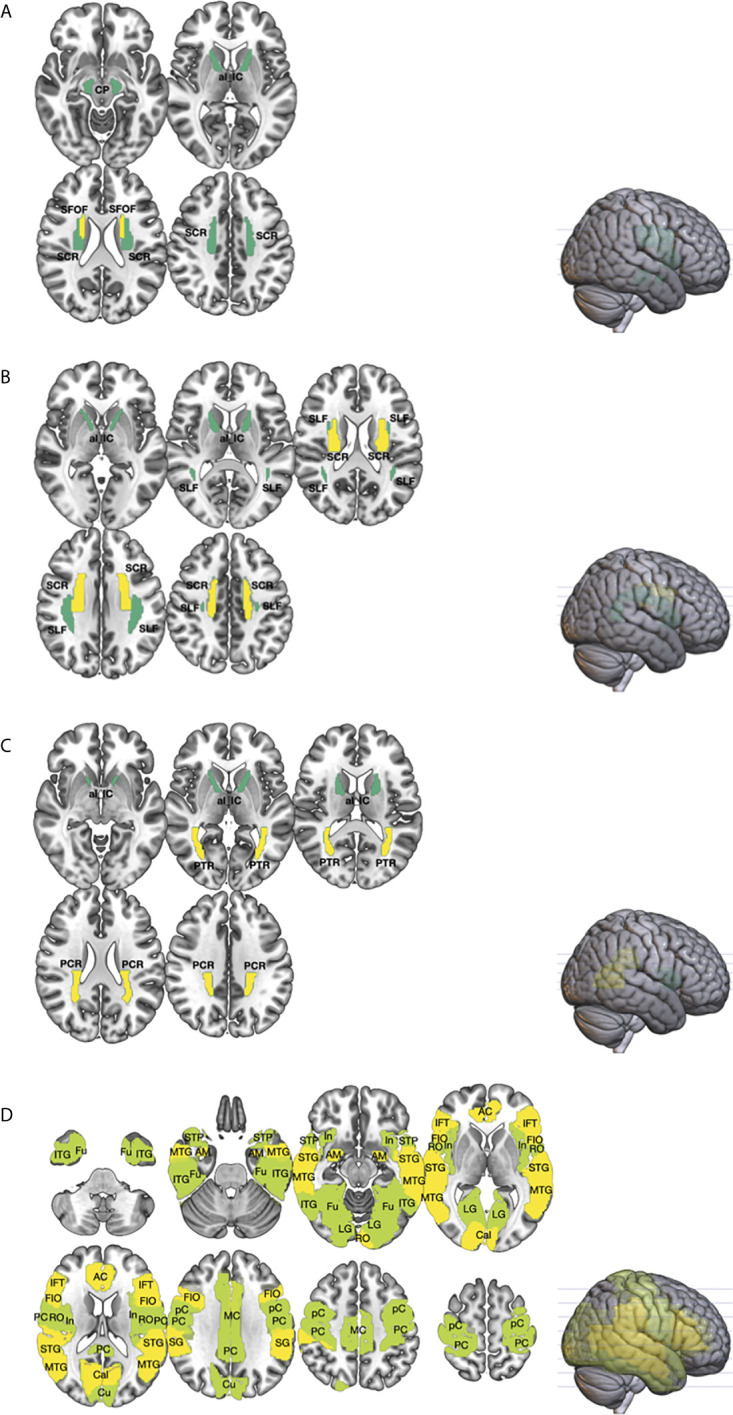
The differences in the white (WM; **(A–C)** and gray matters (GM; **(D)** between germ cell tumor (GCT; n = 20) survivors and healthy age, sex-matched controls (n = 14) were investigated using diffusion metrics, namely mean diffusivity (MD), fractional anisotropy (FA), and mean kurtosis (MK), obtained from diffusional kurtosis imaging (DKI). Region-of-interest (ROI) measurement of the cortical and subcortical regions, from the Automated anatomical labeling (AAL) atlas, and major WM tracts, from the Johns Hopkins white matter atlas, were performed. The brain regions that showed statistically significant difference in diffusion metrics between GCT and healthy controls were shown (*P* < 0.01 in yellow; *P* < 0.05 in green). As compared to healthy controls, ROI analyses of the WM showed that the MD **(A)** of GCT survivors in the cerebral peduncle (CP), superior corona radiata (SCR), anterior limb of internal capsule (al_IC), and superior fronto-occipital fasciculus (SFOF) were higher; the MK **(B)** in the superior longitudinal fasciculus (SLF), SCR, and al_IC were lower; and the FA **(C)** in the posterior corona radiata (PCR), posterior thalamic radiation (PTR), and ac_IC were lower. Region-of-interest analysis of the GM **(D)** showed that the MD of GCT in fusiform (Fu), inferior temporal gyrus (ITG), middle temporal gyrus (MTG), superior temporal pole (STP), superior temporal gyrus (STG), amygdala (AM), insular (In), lingual gyrus (LG), rolandic operculum (RO), anterior cingulum (AC), middle cingulum (MC), posterior cingulum (PC), calcarine (Cal), cuneus (Cu), precentral gyrus (pC), postcentral gyrus (PC), supramarginal gyrus (SG), frontal inferior operculum (FIO), and inferior frontal triangularis (IFT) were significantly higher than those of healthy control.

Compared to healthy controls, GCT survivors had significantly higher MD in the cingulum, fornix, uncinate fasciculus, superior fronto-occipital fasciculus, anterior limb of internal capsule, anterior and superior corona radiata, and the cerebral peduncle (**Figure 1A**, **Supplementary Table 1**). They had significantly lower MK in the cingulum, fornix, superior longitudinal fasciculus, anterior and superior corona radiate, and anterior limb of the internal capsule (**Figure 1B**). They also had significantly lower FA values in the cingulum, fornix, posterior corona radiata, posterior thalamic radiation, and anterior limb of the internal capsule (**Figure 1C**).

### Diffusion Metrics of Grey Matter

Compared to healthy controls, GCT survivors had significantly higher MD in the anterior, middle and posterior cingulum, amygdala, frontal inferior triangularis, Heschl’s gyrus, supramarginal gyrus, calcarine sulcus, superior and middle inferior temporal gyrus, precentral and postcentral gyrus, frontal inferior operculum, superior temporal pole, insula, fusiform, rolandic operculum, lingual gyrus, and cuneus (**Figure 1D**, **Supplementary Table 2**).

### Relations Between Diffusion Metrics of the Whole Brain Versus IQ and KPS Scores

Lower IQ (**Table 2A**) and KPS (**Table 2B**) scores were associated with higher MD values in the white matter of the whole brain. No significant associations were found for the grey matter of the whole brain.

**Table 2a:** Multiple regression analysis of the relationships between IQ and diffusion metrics of the whole brain.

Regions	Effect	95% CI	*P*
**IQ – White matter MD**			
Whole brain	-79.66	(-153.66, -5.65)	0.040
**IQ – White matter MK**			
Whole brain	106.37	(-13.57, 226.32)	0.084
**IQ – Grey matter MD**			
Whole brain	-63.74	(-119.72, -7.75)	0.119

MD, mean diffusivity; MK, mean kurtosis.

**Table 2b:** Multiple regression analysis of the relationships between Karnofsky score and diffusion metrics of the whole brain.

Regions	Effect	95% CI	*P*
**Karnofsky score – White matter MD**			
Whole brain	-84.78	(-149.67, -19.88)	0.017
**Karnofsky score – White matter MK**			
Whole brain	105.27	(-6.44, 216.88)	0.069
**Karnofsky score – White matter FA**			
Whole brain	181.93	(1.37, 362.49)	0.054
**Karnofsky score – Grey matter MD**			
Whole brain	-27.99	(-87.7, 31.73)	0.343

MD, mean diffusivity; MK, mean kurtosis; FA, fractional anisotropy.

### Relations Between IQ Scores and Diffusion Metrics of All Brain Regions in the AAL Atlas

For the white matter of GCT survivors, lower IQ scores were associated with higher MD values in the anterior limb of the internal capsule, superior fronto-occipital fasciculus, anterior corona radiata, uncinate fasciculus, cingulum, and the hippocampus; and lower IQ scores were associated with lower MK values in the superior fronto-occipital fasciculus. For the grey matter of GCT survivors, lower IQ scores were associated with higher MD values in the olfactory cortex, insula, caudate, Heschl’s gyrus, parahippocampal gyrus, hippocampus, anterior cingulum, frontal inferior operculum, middle and superior temporal gyrus, middle and superior frontal orbital gyrus, cuneus, and precentral gyrus. All results are summarized in **Table 3A**.

**Table 3a:** Multiple regression analysis of the relationships between IQ and diffusion metrics of all brain regions in the AAL atlas.

Regions	Effect	95% CI	*P*
**IQ – White matter MD**			
Anterior limb of internal capsule	-71.83	(-31.02, -112.65)	<0.01
Superior fronto-occipital fasciculus	-34.14	(-13, -55.28)	<0.01
Uncinate fasciculus	-56.25	(-18.02, -94.48)	<0.01
Anterior corona radiata	-115.27	(-33.36, -197.19)	<0.05
Cingulum cingulate	-116.68	(-10.15, -223.21)	<0.05
Cingulum hippocampus	-50.34	(-1.74, -98.95)	<0.05
**IQ – White matter MK**			
Superior fronto-occipital fasciculus	73.37	(122.1, 24.64)	<0.01
**IQ – Grey matter MD**			
Heschl’s gyrus	-41.04	(-14.58, -67.5)	<0.01
Insula	-64.02	(-22.2, -105.85)	<0.01
Parahippocampal gyrus	-70.34	(-24.28, -116.39)	<0.01
Caudate	-27.81	(-7.5, -48.13)	<0.05
Olfactory	-42.41	(-10.11, -74.71)	<0.05
Anterior cingulum	-71.1	(-16.75, -125.44)	<0.05
Hippocampus	-29.31	(-5.25, -53.36)	<0.05
Middle frontal orbital gyrus	-58.83	(-9.44, -108.23)	<0.05
Precentral gyrus	-53.16	(-8.26, -98.06)	<0.05
Frontal inferior operculum	-55.91	(-8.24, -103.57)	<0.05
Cuneus	-41.52	(-4.45, -78.6)	<0.05
Superior temporal gyrus	-45.72	(-2.67, -88.77)	<0.05
Superior frontal orbital gyrus	-38.18	(-1.83, -74.54)	<0.05
Middle temporal gyrus	-74.86	(-3.17, -146.55)	<0.05

MD, mean diffusivity; MK, mean kurtosis.

### Relations Between KPS Scores and Diffusion Metrics of All Brain Regions in the AAL Atlas

For the white matter of GCT survivors, KPS scores were negatively correlated with MD and positively correlated with FA and MK in the cerebral peduncle, superior longitudinal fasciculus, external capsule, and posterior limb of the internal capsule. Similar associations between KPS scores and MD were found in the superior and posterior corona radiata, and retrolenticular part of the internal capsule. Similar associations between KPS scores and MK were found in the posterior and superior corona radiata, uncinate fasciculus, and superior fronto-occipital fasciculus. For the grey matter of GCT survivors, KPS scores were negatively correlated with MD in the pallidum, putamen, thalamus, rolandic operculum, and inferior temporal gyrus. All results are summarized in **Table 3B**.

**Table 3b:** Multiple regression analysis of the relationships between Karnofsky score and diffusion metrics of all brain regions in the AAL atlas.

Regions	Effect	95% CI	*P*
**Karnofsky score – White matter MD**			
Cerebral peduncle	-40.59	(-15.8, -65.38)	<0.01
Superior corona radiata	-84.7	(-28.04, -141.37)	<0.01
Posterior limb of internal capsule	-47.57	(-13.94, -81.2)	<0.01
Posterior corona radiata	-69.64	(-17.34, -121.94)	<0.05
Retrolenticular part of internal capsule	-50.61	(-12.44, -88.78)	<0.05
External capsule	-54.93	(-9.4, -100.46)	<0.05
Superior longitudinal fasciculus	-67.26	(-9.26, -125.27)	<0.05
**Karnofsky score – White matter MK**			
Superior longitudinal fasciculus	96.26	(173.9, 18.61)	<0.05
Cerebral peduncle	66.64	(122.41, 10.87)	<0.05
Posterior corona radiata	91.83	(169.67, 13.98)	<0.05
External capsule	127.46	(237.96, 16.97)	<0.05
Superior corona radiata	83.21	(155.57, 10.84)	<0.05
Uncinate fasciculus	94.75	(179.01, 10.48)	<0.05
Posterior limb of internal capsule	58.17	(112.56, 3.78)	<0.05
Superior fronto-occipital fasciculus	47.1	(91.38, 2.82)	<0.05
**Karnofsky score – White matter FA**			
Retrolenticular part of internal capsule	143.8	(233.52, 54.08)	<0.01
External capsule	175.26	(295.97, 54.55)	<0.01
Posterior limb of internal capsule	96.49	(172.27, 20.72)	<0.05
Uncinate fasciculus	111.13	(199.23, 23.02)	<0.05
Cerebral peduncle	97.69	(175.68, 19.7)	<0.05
Posterior thalamic radiation	156.4	(284.46, 28.34)	<0.05
Superior longitudinal fasciculus	168.56	(308.32, 28.79)	<0.05
Fornix	106.65	(196.4, 16.9)	<0.05
**Karnofsky score – Grey matter MD**			
Pallidum	-37.01	(-9.83, -64.2)	<0.05
Putamen	-57.12	(-11.58, -102.66)	<0.05
Thalamus	-39.31	(-7.17, -71.45)	<0.05
Rolandic operculum	-38.88	(-2.93, -74.82)	<0.05
Inferior temporal gyrus	-72.11	(-1.85, -142.37)	<0.05

MD, mean diffusivity; MK, mean kurtosis; FA, fractional anisotropy.

## Discussion

We have successfully demonstrated using diffusional kurtosis imaging that there were extensive microstructural changes in the grey and white matter of intracranial GCT survivors with a history of cranial irradiation when compared to healthy controls. The majority of these changes occurred in brain regions related to cognition and motor functions. Our study provides evidence that these extensive microstructural changes might underlie deficits in cognitive and motor functions, as well as problems with sensory processing. Our study also demonstrated that it is important to look at the microstructural changes of individual brain regions rather than just looking at the whole brain. Brain regions affected by the tumor or focal radiotherapy will be prone to more severe microstructural damages. Therefore, despite of the lack of association between IQ or KPS scores and microstructural changes in the whole brain grey matter, we found significant associations between the microstructural changes of specific grey matter regions and the IQ and KPS scores, which reflect cognitive and functional outcomes respectively.

A recent study showed that childhood leukemia survivors with history of cranial radiotherapy had altered diffusion metrics in the fornix, uncinate fasciculus, and cingulum, which are important structures that subserve episodic memory, learning, and attention (22). Leung et al. found significant change in the FA of the posterior thalamic radiation of medulloblastoma survivors after cranial irradiation and chemotherapy (23). We found GCT survivors had significant microstructural changes in these brain regions, as well as in projection fibers such as the corona radiata, posterior thalamic radiation, and superior longitudinal fasciculus. Furthermore, significant changes were detected in the anterior limb of the internal capsule containing projection fibers between the lentiform nucleus and caudate nucleus, which are responsible for the control of motor and sensory pathways.

It is well known that executive function, attention and memory are regulated by deep brain nuclei in the subcortical regions of the brain. We found significant microstructural changes in the grey matter of intracranial GCT survivors. Significant differences were found in the MD of grey matter regions responsible for language, cognition, and executive functions, including the cingulum, amygdala, temporal gyrus, fusiform, and rolandic operculum. Significant differences were also found in brain regions responsible for visual processing, including the calcarine, lingual gyrus, and cuneus. Other affected brain regions included Heschl’s gyrus, which is the primary auditory cortex, the precentral gyrus containing the primary motor cortex, and the postcentral gyrus containing the somatosensory cortex.

The estimated FSIQ is typically derived from subtests for performance IQ as well as verbal IQ. Our results showed that the IQ scores were negatively correlated with the MD of brain regions responsible for language processing, including those related to Wernicke’s area in the temporal lobe. Heschl’s gyrus is associated with the processing of speech-related cues, which facilitates learning and perception of new speech sounds (24). In addition, lower IQ scores were found to be associated with the MD of brain regions responsible for cognitive function such as the hippocampus, parahippocampal gyrus, and anterior cingulum. The frontal regions of the brain are known to be involved in cognitive functions and control of emotions. Our study showed significant association between FSIQ scores and MD values in frontal regions such as the orbitofrontal cortex as well as regions around Broca’s area. The frontal inferior opercularis acts indirectly through the motor cortex within the precentral gyrus to control the motor aspects of speech production (25). Interestingly, it is common for patients with neurodegenerative diseases such as Alzheimer’s disease and dementia to have motor speech deficits (26, 27). Hence, GCT survivors might be prone to motor speech problems. Our findings are consistent with previous studies on childhood acute lymphoblastic leukemia survivors with a history of chemotherapy and/or radiotherapy, which found that lower volume of the caudate nucleus, was associated with significantly worse verbal fluency (28). In addition, our study demonstrated the significant association of the precentral gyrus with the IQ scores of GCT survivors, suggestive of the involvement of the precentral gyrus in cognitive tasks. The precentral gyrus is the site of the primary motor cortex and is traditionally implicated in voluntary movement control. However, more recent studies suggested that the primary motor cortex not only plays a role in stimulus-response compatibility, plasticity, motor sequence learning and memory as well as learning of sensorimotor associations, but is also engaged in motor imagery, spatial transformations and working memory tasks (29–32). Therefore, our study provides additional evidence to support the involvement of the primary motor cortex in higher cognitive tasks.

Most of the microstructural changes that were associated with functional impairment, as indicated by the poor KPS score, were in white matter regions. These regions included the pyramidal tracts within the internal capsule or fiber tracts such as the corona radiata above the basal ganglia, which had elevated MD but reduced MK and FA. Our findings were consistent with studies on children with cerebral palsy, which found significant correlation between motor function scores and the FA of sensory and motor pathways such as the corticospinal tract, thalamic radiation, or the posterior limb of the internal capsule (33). Functional impairments that were associated with microstructural changes in grey matter regions were mostly confined around the basal ganglia, which have important roles in motor control.

Historically, radiation damage was thought to affect brain white matter rather than the cortex itself (12, 34). Nevertheless, a recent study demonstrated that brain tumor survivors with a history of irradiation had cortical atrophy that was dependent on the radiation dose (35). Seibert et al. showed that cortical atrophy in patients after brain radiotherapy was significantly associated with radiation dose in the entorhinal and inferior parietal regions, which are areas responsible for memory and executive functions (36). They also demonstrated that these cerebral cortex regions seemed to be more vulnerable to dose-dependent radiation atrophy. Our study corroborates the findings by Seibert et al. We also found significant correlations between grey matter microstructural changes and cognitive function in GCT survivors with history of brain radiotherapy. Our study is first to demonstrate significant associations between white and grey matter microstructural changes in vulnerable brain regions of GCT survivors in association with cognitive impairments and/or functional deficits.

Our study had several limitations. First, the number of study subjects was small. Nevertheless, our study was the first to perform DKI measurements in association with cognitive and physical functioning assessments in a cohort of GCT survivors. In addition, our study participants were predominantly male (80%). However, predilection to the male gender is a well-known characteristic of GCT (37) and previous studies also demonstrated similar male: female ratio (38, 39). Second, due to the retrospective nature of the study, we did not have information on the physical or neurocognitive functions in our cohort at the time of tumor diagnosis. Therefore, it is hard to ascertain if the cognitive or functional impairments are due to individual or combined effects of the tumor, chemotherapy, or radiotherapy. Nevertheless, radiation-induced brain damage has long been recognized in pediatric cancer patients, more recent studies have shown that chemotherapy can also lead to neurotoxicity (40). The microstructural brain changes and cognitive or functional impairments that were found in the current study are likely due to the combined effects of all three factors. Lastly, the measurement of the diffusion metrics of GM regions could be confounded by the partial volume effect from free fluid such as cerebral spinal fluid, for which could not be accounted in our statistical analyses.

In conclusion, our DKI study has successfully demonstrated that impairment in cognitive function and/or deficits in physical functioning were associated with microstructural changes in multiple white and grey matter regions. Proton radiotherapy with reduced irradiation dosage to vulnerable brain regions might lead to improved cognitive and functional outcomes. Diffusion metrics obtained from DKI could potentially be used to identify patients at high risk of cognitive or functional impairment for timely interventions.

## Data Availability Statement

The raw data supporting the conclusions of this article will be made available upon request to the authors.

## Ethics Statement

The studies involving human participants were reviewed and approved by The Institutional Review Board of the University of Hong Kong/Hospital Authority Hong Kong West Cluster (HKU/HA HKW IRB), the Research Ethics Committee of the Kowloon Central/ Kowloon East Cluster (KC/KE CREC), Kowloon West Cluster (KW CEREC) and New Territories West Cluster (NTW CEREC). Written informed consent to participate in this study was provided by the participants’ legal guardian/next of kin.

## Author Contributions

Conceptualization: WT, EH, TL, PK and GC. Data curation: WT, EH, TL, AL, PI, VV, KC, DF, DHFC, FH, KY, DK, DKLC, CL, MS, LL, PK and GC. Formal analysis: WT, EH, TL, AL, KC, DF, FH, KY, LL, PK and GC. Funding acquisition: WT. Investigation: WT, EH, TL, VV, PK and GC. Methodology: WT, EH, TL, AL, PI and FH, PK and GC. Project administration: WT, EH, TL, PK and GC. Software: EH and KY. Supervision: WT, EH, TL, PK and GC. Validation: DF. Writing – original draft: WT and EH. Writing – review and editing: WT, EH, TL, AL, PI, VV, KC, DF, DHFC, FH, KY, DK, DKLC, CL, MS, LL, PK and GC. All authors contributed to the article and approved the submitted version.

## Funding

This work was supported by Research Grants Council of the Hong Kong Special Administrative Region, China (No. HKU 17118815). The funder had no role in the design of the study; the collection, analysis, and interpretation of the data; the writing of the manuscript; and the decision to submit the manuscript for publication.

## Conflict of Interest

The authors declare that the research was conducted in the absence of any commercial or financial relationships that could be construed as a potential conflict of interest.
