# Supplementary material for: Brain Microstructural Changes Associated With Neurocognitive Outcome in Intracranial Germ Cell Tumor Survivors
Source: Front Oncol. 2021 May 26;11:573798. doi: 10.3389/fonc.2021.573798 (PMC8216078; doi:10.3389/fonc.2021.573798)
Supplement: Supplementary file 1 [file Table_1.docx]

**Supplementary Tables**

**Table 1**. White matter regions that show significance difference in diffusion metrics between the 20 GCT survivors and 14 healthy age and sex-matched controls

| **Regions** | **Estimate, *β*** | **SD** | ***P*** |
| --- | --- | --- | --- |
| **MD** |  |  |  |
| Anterior corona radiata | 0.07 | 0.02 | <0.01 |
| Superior fronto-occipital fasciculus | 0.18 | 0.07 | <0.01 |
| Cingulum | 0.04 | 0.02 | <0.05 |
| Anterior limb of internal capsule | 0.08 | 0.03 | <0.05 |
| Uncinate fasciculus | 0.09 | 0.04 | <0.05 |
| Fornix | 0.15 | 0.06 | <0.05 |
| Superior corona radiata | 0.05 | 0.02 | <0.05 |
| Cerebral peduncle | 0.1 | 0.05 | <0.05 |
| **MK** |  |  |  |
| Superior corona radiata | -0.05 | 0.02 | <0.01 |
| Fornix | -0.05 | 0.02 | <0.01 |
| Anterior corona radiata | -0.05 | 0.02 | <0.05 |
| Anterior limb of internal capsule | -0.06 | 0.02 | <0.05 |
| Cingulum | -0.04 | 0.01 | <0.05 |
| Superior longitudinal fasciculus | -0.04 | 0.02 | <0.05 |
| **FA** |  |  |  |
| Fornix | -0.06 | 0.02 | <0.01 |
| Posterior thalamic radiation | -0.04 | 0.01 | <0.01 |
| Cingulum | -0.05 | 0.02 | <0.01 |
| Posterior corona radiata | -0.03 | 0.01 | <0.01 |
| Anterior limb of internal capsule | -0.03 | 0.01 | <0.05 |

White matter regions. MD: mean diffusivity, MK: mean kurtosis, FA: fractional anisotropy

**Table 2**. Grey matter regions that show significance difference in diffusion metrics between the 20 GCT survivors and 14 healthy age and sex-matched controls

| **Regions** | **Estimate, *β*** | **SD** | ***P*** |
| --- | --- | --- | --- |
| **MD** |  |  |  |
| Anterior cingulum | 0.11 | 0.03 | <0.01 |
| Inferior frontal triangularis | 0.1 | 0.03 | <0.01 |
| Heschl’s gyrus | 0.18 | 0.05 | <0.01 |
| Supramarginal gyrus | 0.09 | 0.03 | <0.01 |
| Amygdala | 0.16 | 0.05 | <0.01 |
| Calcarine | 0.09 | 0.03 | <0.01 |
| Superior temporal gyrus | 0.1 | 0.04 | <0.01 |
| Middle temporal gyrus | 0.06 | 0.02 | <0.01 |
| Frontal inferior operculum | 0.09 | 0.03 | <0.01 |
| Precentral gyrus | 0.08 | 0.03 | <0.05 |
| Middle cingulum | 0.08 | 0.03 | <0.05 |
| Insula | 0.08 | 0.03 | <0.05 |
| Inferior temporal gyrus | 0.05 | 0.02 | <0.05 |
| Fusiform | 0.06 | 0.03 | <0.05 |
| Posterior cingulum | 0.06 | 0.03 | <0.05 |
| Postcentral gyrus | 0.08 | 0.03 | <0.05 |
| Rolandic operculum | 0.08 | 0.04 | <0.05 |
| Superior temporal pole | 0.13 | 0.06 | <0.05 |
| Lingual gyrus | 0.06 | 0.03 | <0.05 |
| Cuneus | 0.09 | 0.04 | <0.05 |

Grey matter regions. MD: mean diffusivity
